# Ultrafast CMR to deliver high volume screening of an at risk thalassemia population in the developing world: preliminary results from the TIC-TOC study (Thailand and UK international collaboration in thalassaemia using an optimised ultrafast CMR protocol)

**DOI:** 10.1186/1532-429X-18-S1-O39

**Published:** 2016-01-27

**Authors:** Amna Abdel-Gadir, Yongkasem Vorasettakarnkij, Hataichanok Ngamkasem, Sabrina Nordin, Emmanuel O Ako, Monravee Tumkosit, Pranee Sutcharitchan, Peter Kellman, Stefan K Piechnik, Juliano L Fernandes, Mark Westwood, John Porter, John Malcolm Walker, James Moon

**Affiliations:** 1 Institute of Cardiovascular Science, University College London, London, UK; 2 Barts Heart Centre, London, UK; 3 Chulalongkorn University, Bangkok, UK; 4 NIH, Bethesda, MD USA; 5 University of Oxford, Oxford, UK; 6 Jose Michel Kalaf Research Institute, Sao Paulo, Brazil; 7 Haematology, University College London, London, UK

## Background

The vast majority (>100,000) of thalassemia patients at risk of iron overload live in countries with limited red cell transfusions and chelating agents, with serum ferritin used as the method for iron monitoring. Cardiac iron can cause heart failure, but iron detection using CMR is perceived as expensive, time consuming and difficult. Parametric mapping (T2* or T1) can be fast and allows instant recognition of iron loading.

We aimed to perform CMR in the developing world to quantify cardiac and liver iron, assessing its speed, cost, reliability, and clinical information yield.

## Methods

**Figure 1:**
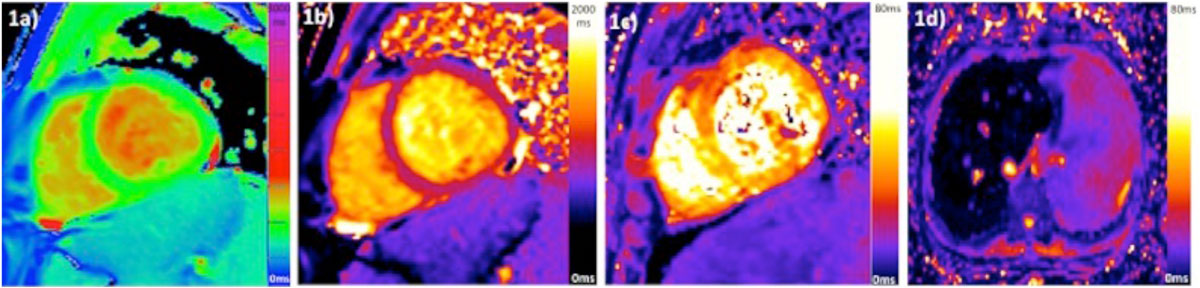
T1 and T2* maps acquired. 1a) myocardial ShMOLLI; 1b) myocardial MOLLI; 1c) myocardial T2*; 1d) liver T2*. Liver T1 values were obtained from the short axis views

In a leading government hospital in Bangkok, Thailand we set up an ultrafast mapping protocol and analysis pipeline. In 2 days, 128 scans were performed in 97 thalassaemia patients and 11 healthy volunteers. The protocol consisted of: localisers, HASTE, pilots, T2* and T1 maps (figure 1a,b,c,d), and 2 and 4 chamber cines. A short axis stack was also acquired if there was evidence of impairment on long axis cine imaging. Maps were analysed immediately and off-line (truth standard). Repeatability was performed in 20 (10 patients and 10 healthy volunteers). Thalassemia subtype, transfusion and past medical history, mean and same day ferritin levels served as comparators.

## Results

Baseline patient characteristics are shown in table 1. The mean patient age was 34 ± 12 (70% female). 64 scans per day were performed with a mean of 6 patients per hour, and 8.3 ± 2.4 minutes per scan. Analysis of T1 and T2* maps was completed within 1 minute of last image acquisition. 91% of patients had liver iron by T2* comprising of 41 (42%) with severe loading, 29 (30%) moderate, 18 (19%) mild, and 9 (9%) undetectable. 15 (16%) of patients had cardiac iron by T2*: 3 mild, 2 moderate, and 10 severe. Instant analysis of T1 and T2* maps was robust with high concordance with traditional off-line analysis. In the heart, T1 mapping agreed with T2* with excellent correlation (T2* vs myocardial ShMOLLI r = 0.885; vs MOLLI r = 0.875, both p < 0.0001). However, 24%(23) additional patients had normal myocardial T2* but low T1 values, suggesting missed iron. In the liver, T1 mapping agreed with T2* (ShMOLLI r = 0.598; MOLLI r = 0.582, both p < 0.0001), and correlated with mean ferritins (p < 0.005). T1 mapping had higher correlation with ferritin than T2*, particularly in severe patients. However, in most patients the T2* curves were censored to just 2 points.

**Table 1:** Patient baseline characteristics.

N	97
Male/ Female	31/66
Age (years)	34.1 ± 12.1
Thalassemia subtype (%)	
Thal/ HbE	60
Thalassemia intermedia	29
Thalassemia major	11
Transfusion history (%)	
None	4
Less than 8 units/yr	4
Greater than 8 units/yr	92
Hb (g/dL)	8.05 ± 1.24
Ferritin (ug/L)	3533 ± 2897
Scan duration (mins)	8.3 ± 2.4

## Conclusions

Ultrafast CMR in the developing world using parametric mapping for iron overload is possible. We have demonstrated that 60 scans a day at 8 minutes per scan is achieveable. A follow-up study and pilot clinical service is underway.

